# Supplementary material for: Targeted Resequencing of Otosclerosis Patients from Different Populations Replicates Results from a Previous Genome-Wide Association Study
Source: J Clin Med. 2022 Nov 26;11(23):6978. doi: 10.3390/jcm11236978 (PMC9737413; doi:10.3390/jcm11236978)
Supplement: Supplementary file 1 [file jcm-11-06978-s001.zip › jcm-2028914-supplementary.pdf]

Supplementary Table S1 Cases and controls included in this study

The table gives an overview of the number of cases and controls used in this study. Numbers are given for the total population and all subpopulations. For the total population mean age at time of collection was calculated and percentages of female and male participants are given.

**Total population (N cases/controls)**	**1696 /1584**
**Mean age at collection (SD)**	47.69 (13.34)
**Gender (% female/male)**	60.86/39.13
**Belgian (N cases/controls)**	796/854
**Dutch (N cases/controls)**	143/118
**French (N cases/controls**	347/367
**Italian (N cases/controls)**	229/63
**Romanian (N cases/controls)**	87/89
**Swiss (N cases/controls)**	94/93

Supplemental Table S2 Result of Linkage Disequilibrium between variants

Linkage disequilibrium (LD) between variants was calculated using the Linkage Dise-quilibrium Calculator of Ensembl (EMBL-EBI, Hinxton, UK) using data from the 1000 Ge-nomes Project database. Variants occuring in the same gene are used for calculations. Each line gives the r^2 and D' between two variants.

**Gene**	**Variant 1**	**Variant 2**	**r^2**	**D'**
**AHSG**	rs4917	rs4918	1.000000	1.000000
	rs4918	rs1071592	0.769229	0.999999
	rs4917	rs1071592	0.769229	0.999999
**LINC01482**	rs3744501	rs8070086	0.397106	0.717946
	rs3744501	rs2278445	0.111645	0.653026
	rs3744501	rs34216978	0.100347	0.999975
	rs34216978	rs11868207	0.057586	0.853398
	rs2278445	rs11868207	0.206276	0.999982
	rs2278445	rs8070086	0.201689	0.999976
	rs34216978	rs8070086	0.055878	0.850148

	rs11868207	rs8070086	0.977773	1.000000
	rs3744501	rs11868207	0.380730	0.695129
**MARK3**	rs1951391	rs2273699	0.978346	1.000000
	rs1951391	rs13987	0.371420	0.999988
	rs11541718	rs1951391	0.371420	0.999988
	rs2273699	rs13987	0.363378	0.999990
	rs11541718	rs13987	1.000000	1.000000
	rs11541718	rs2273699	0.363378	0.999990
**RELN**	rs39335	rs39374	0.094318	0.846650
	rs39335	rs3914132	0.644475	0.955869
	rs39395	rs7791481	0.182095	0.999987
	rs39352	rs7791481	0.094847	0.999946
	rs2299383	rs39335	0.280605	0.999986
	rs39374	rs3914132	0.186535	0.999981
	rs39350	rs7791481	0.064219	0.999899
	rs39335	rs39352	0.093591	0.852162
	rs2299383	rs39350	0.274041	0.938694
	rs39374	rs39395	0.463093	0.933161
	rs2299383	rs7791481	0.163365	0.889380
	rs39335	rs39395	0.211247	0.924015
	rs39350	rs39395	0.319325	0.951471
	rs39335	rs39350	0.087260	0.999932
	rs39352	rs39374	0.979503	1.000000
	rs39374	rs7791481	0.096834	0.999958
	rs39352	rs3914132	0.182706	0.999965

	rs39395	rs3914132	0.350767	0.999996
	rs39350	rs39352	0.677143	0.999999
	rs2299383	rs39395	0.182891	0.455443
	rs39350	rs3914132	0.123717	0.999960
	rs2299383	rs3914132	0.285873	0.847692
	rs39350	rs39374	0.663263	0.999998
	rs39352	rs39395	0.486350	0.966260

Supplemental Table S3 Result of gene-based tests

Gene-based tests were carried out under a wide range of models and assumptions, stratifying for variant type. Gene-based tests included three mutation burden tests (Combined and Multivariate collapsing test (CMC), kernel-based adaptive cluster (KBAC) test, and the Variable Thresholds method (VT)), and one variance component analyses (cAlpha test). Variant type "All" represents all very rare variants with a minor allele frequency (MAF) smaller than 0.01. All other variant types are the results of stratifying for variant. For each test the uncorrected p-value is provided with the corresponding q-value after multiple testing between brackets.

**Gene**	**Variant type**	**Mutation burden tests**			**Variance component analysis**
		**CMC**	**KBAC**	**VT**	**cAlpha**
AHSG	All	0.64 (0.94)	0.33 (0.75)	0.46 (0.79)	0.69 (0.96)
	Intronic	1 (1)	0.32 (0.75)	0.31 (0.75)	0.82 (0.99)
	Exonic	0.32 (0.75)	0.16 (0.59)	0.19 (0.59)	0.53 (0.85)
	Non-synonymous & Frameshift	*	*	*	*
	3'-UTR	0.15 (0.62)	1 (1)	1 (1)	0.60 (0.91)
	5'-UTR	0.75 (0.96)	0.35 (0.75)	0.44 (0.78)	0.87 (1)
EYA2	All	0.61 (0.92)	0.18 (0.62)	0.34 (0.75)	0.53 (0.87)

	Intronic	1 (1)	0.49 (0.83)	0.40 (0.75)	0.83 (0.99)
	Exonic	0.28 (0.75)	0.052 (0.44)	0.095 (0.53)	0.53 (0.85)
	Non-synonymous & Frameshift	**0.022** (0.31)	**0.004** (0.16)	**0.027** (0.34)	0.064 (0.48)
	3'-UTR	0.11 (0.60)	0.97 (1)	0.98 (1)	0.18 (0.62)
	5'-UTR	*	*	*	*
LINC01482	All	**0.030** (0.34)	**0.0064** (0.16)	0.061 (0.48)	**0.0060** (0.16)
	Intronic	0.18 (0.62)	**0.034** (0.34)	0.31 (0.75)	**0.015** (0.30)
	Exonic	*	*	*	*
	Non-synonymous & Frameshift	*	*	*	*
	3'-UTR	*	*	*	*
	5'-UTR	*	*	*	*
MARK3	All	1 (1)	0.44 (0.78)	0.24 (0.70)	0.57 (0.88)
	Intronic	0.092 (0.56)	0.82 (0.99)	0.23 (0.70)	0.15 (0.62)
	Exonic	0.91 (1)	0.53 (0.85)	0.64 (0.92)	0.76 (0.94)
	Non-synonymous & Frameshift	1 (1)	0.53 (0.86)	0.73 (0.96)	0.39 (0.75)
	3'-UTR	0.50 (0.83)	0.31 (0.75)	0.36 (0.75)	0.84 (0.99)
	5'-UTR	0.15 (0.62)	0.067 (0.48)	0.088 (0.55)	0.38 (0.75)
RELN	All	0.56 (0.88)	0.29 (0.75)	0.52 (0.84)	0.55 (0.87)
	Intronic	0.13 (0.61)	0.052 (0.45)	**0.035** (0.34)	0.066 (0.48)
	Exonic	0.96 (1)	0.56 (0.87)	0.84 (0.97)	0.66 (0.93)
	Non-synonymous & Frameshift	0.56 (0.88)	0.62 (0.93)	0.56 (0.88)	0.78 (0.97)
	3'-UTR	*	*	*	*
	5’-UTR	1 (1)	0.28 (0.75)	0.16 (0.62)	0.69 (0.96)
SUPT3H	All	0.92 (1)	0.38 (0.74)	0.34 (0.74)	0.28 (0.74)

	Intronic	0.73 (0.96)	0.35 (0.75)	0.32 (0.75)	0.94 (1)
	Exonic	0.10 (0.58)	**0.026** (0.32)	0.18 (0.59)	0.089 (0.52)
	Non-synonymous & Frameshift	*	*	*	*
	3'-UTR	0.19 (0.62)	0.95 (1)	0.80 (0.97)	0.71 (0.96)
	5'-UTR	1 (1)	0.37 (0.75)	0.31 (0.75)	0.73 (0.96)
TGFβ1	All	0.37 (0.74)	0.16 (0.59)	0.086 (0.52)	0.43 (0.77)
	Intronic	1 (1)	0.57 (0.88)	0.76 (0.96)	0.76 (0.96)
	Exonic	0.36 (0.74)	0.088 (0.52)	0.20 (0.60)	0.38 (0.74)
	Non-synonymous & Frameshift	0.13 (0.60)	**0.020** (0.31)	**0.041** (0.39)	0.41 (0.76)
	3'-UTR	0.72 (0.96)	0.69 (0.96)	0.66 (0.96)	0.78 (0.97)
	5'-UTR	0.39 (0.75)	0.12 (0.60)	**0.0064** (0.16)	0.13 (0.61)

* The number of variants was insufficient to carry out the gene-based tests
